# High Pulsed Voltage Alkaline Electrolysis for Water Splitting

**DOI:** 10.3390/s23083820

**Published:** 2023-04-08

**Authors:** Matías Albornoz, Marco Rivera, Patrick Wheeler, Roberto Ramírez

**Affiliations:** 1 Department of Electrical Engineering, Faculty of Engineering, Campus Curicó, Universidad de Talca, Merced 437, Curicó 3341717, Chile; 2 Department of Electrical and Electronic Engineering, Faculty of Engineering, University of Nottingham, Nottingham NG7 2RD, UK

**Keywords:** electrolysis, high-voltage pulsed electrolysis, hydrogen production, plasmolysis, pulsed power, water electrolysis

## Abstract

Pulsed electrolysis has become a promising research topic in recent decades due to advances in solid-state semiconductor devices. These technologies have enabled the design and construction of simpler, more efficient, and less costly high-voltage and high-frequency power converters. In this paper, we study high-voltage pulsed electrolysis considering variations in both power converter parameters and cell configuration. Experimental results are obtained for frequency variations ranging from 10 Hz to 1 MHz, voltage changes from 2 V to 500 V, and electrode separations from 0.1 to 2 mm. The results demonstrate that pulsed plasmolysis is a promising method for decomposing water for hydrogen production.

## 1. Introduction

The most common technology used at an industrial level in the decomposition of water to produce hydrogen by electrolysis is based on the application of high currents at low voltages to an electrolyte composed of water with additives, a method called alkaline electrolysis. To obtain higher yields, it is necessary to increase the size of the generating device, raising the cost. In order to make hydrogen production more accessible, it is necessary to make the process compatible with smaller energy sources, such as residential PV systems or car alternators as well as making the equipment cheaper [1,2,3,4].

This paper proposes the optimization of hydrogen generation using contemporary methods evolved from conventional DC electrolysis with the use of solid-state devices. Pulsed power is a promising technology that has been widely studied in electrolysis, allowing for improvements in efficiency. Pulse power can be used for the development of plasma formation [5]. This technology allows for greater control in the reaction chemistry by modulating the frequency and duration of the applied pulses. The study of pulsed power has grown in recent decades because of the exponential improvements in solid-state semiconductor devices [6]. These emerging semiconductor devices allow for the implementation of smaller circuits at a lower cost, increasing the power density when compared to previous hardware.

High voltage discharges can occur during the electrolysis process. These discharges can generate an electric field that is capable of breaking the conductivity limit in electrolytes. When this limit is exceeded, an electric arc is produced as the charge finds the route of conduction with the least resistance. This, in addition to being used in plasma generators, has possible applications in purification and decontamination of water [7,8]. In experiments with plasma electrolysis [9,10], significant increases in gas production efficiency have been achieved.

Other electrochemical processes can potentially increase the efficiency of hydrogen production in a slightly different way [11,12], for example through the use of bioelectrochemical electrolysis. In this method, the decomposition of water via electrolysis is used to obtain hydrogen peroxide ($H_{2}O_{2}$) in Microbial Electrolysis Cells (MEC), implemented as Microbial Fuel Cells (MFC) with an external power source. The chemical obtained from this method is used as an oxidizing agent. This process can also be improved with the application of pulsed power technology.

In this paper, a practical approach to electrolysis is considered using the molecular dynamics of water under the application of pulsed voltage to break its conductivity limit for the formation of plasmolysis. First, the theory and current development of the technologies used are reviewed, then a system for monitoring the process is described and an analysis of the results is presented. This work is based on the intention of reducing energy and implementation costs of hydrogen production, in order to bring it closer to local communities as an accessible alternative fuel.

## 2. State of the Art

### 2.1. Electrolysis

Faraday’s first law of electrolysis postulates that the amount of gas produced in the reaction is directly proportional to the current applied to the electrolyte. In the case of hydrogen production, the current is usually applied to water.

Equation (1) relates the mass of the produced substance *m*, to the total charge circulated by the electrolyte *Q*, the molar mass of the substance *M*, the Faraday constant *F* and the valence number of the substance as an ion in the solution *z*.

(1) $$m=\frac{Q}{F}\frac{M}{z}.$$

**Figure 1:**
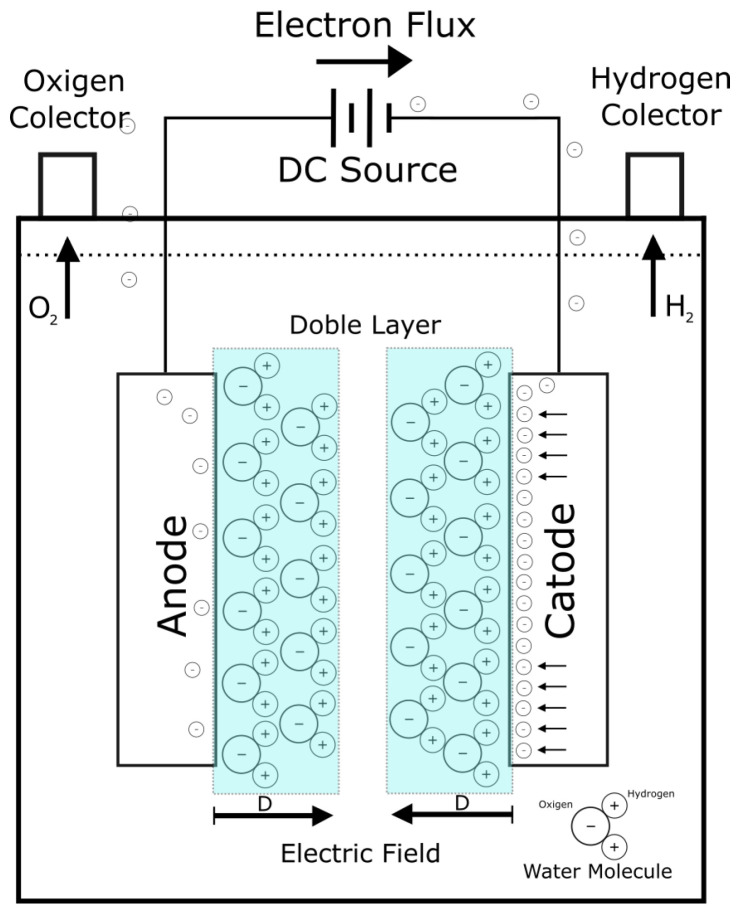
Electrolysis cell and formation of the double layer proportional to the electric field.

The electrolyte container where the electrodes are placed is called the electrolysis cell (Figure 1), inside of it are two electrodes, an anode and a cathode, connected to a DC power supply. Oxygen is generated on the surface of the anode while hydrogen is generated on the surface of the cathode. For the specific situation considered in this paper, when the electrolyte to which an electric current is applied is water, the decomposition voltage is 1.23 V at room temperature (reversible voltage), and an overvoltage of approximately 0.4 V must also be applied. This additional voltage depends on the material of the electrodes [13].

The voltage needed for the electrolysis of water therefore starts at around 1.63 V (thermo-neutral voltage [14]).

To obtain a higher production of hydrogen it is necessary to increase the amount of current applied to the electrolysis cell. If the voltage is increased for this purpose, there is a limit to the energy efficiency, caused by the double capacitive layer effect of water, in addition to the ohmic losses [15,16,17]. If a voltage higher than the reversible voltage is applied, the additional power will be dissipated as heat because of the internal resistance of the cell. The reversible voltage refers to the voltage obtained in the reverse process, when electricity is obtained from the union of hydrogen and oxygen molecules, therefore it is the energy used entirely in the hydrogen production process and that is not wasted as heat. Knowing this principle, electrolysis cells currently used in industry work on continuous voltages that do not exceed 2 V per cell, to ensure maximum efficiency [13].

To regulate the current circulating in the electrolyte, it is possible to modify the conductivity of the water by incorporating additives in the electrolyte solution. The electrolyte can be modified using alkaline additives such as sodium hydroxide (NaOH) or potassium hydroxide (KOH) [9]. The latter shows better results. Thus, it is possible to increase the power and production of hydrogen whilst avoiding the traditional limits.

### 2.2. Pulsed Electrolysis

At the instant that the voltage between the electrodes is applied, an internal movement begins in the molecules of the electrolyte. The polar molecules of the water become positioned in such a way that their electromagnetic orientations are ordered following the electric field between the electrodes.

Before decomposing, the hydrogen atoms of the molecules tend towards the cathode, and the oxygen atoms are located at the other end of the molecule. The same phenomenon occurs on the surface of the anode, only in the reverse situation. This effect is called double layer behavior [15,18] and it gives capacitor qualities to the electrolyzer. When this double layer is formed, the electrons coming from the source are limited in their passage to the electrolyte by the opposite charges present in the molecules; these are ordered causing an electric charge barrier proportional to the voltage applied on the surface of the electrode. Applying high voltages to the electrodes causes a greater internal resistance of the electrolyte, which explains the lower efficiency in these conditions.

This particular behavior of water takes a few milliseconds to form. For this reason, if instead of a DC power source, the voltage is applied as pulses with a sort duration [5,19], it is possible to obtain improvements in the overall efficiency. With voltage pulses in the order of nanoseconds, the current is applied to the cell in such a way that molecular formation which prevents the free flow of electrons is avoided, for an instant of time these are able to circulate through the electrolyte without the opposition of the double layer. The electrons are injected into the electrolyte and it is “charged” with chemically stored energy that is used in the decomposition of water in the time after the pulse. The capacitive behavior of the cell, against a pulsed voltage with a specific frequency, can be modeled as an equivalent RC circuit [15]. It is possible to find a natural frequency for the system [20]. At this natural frequency, there are reductions in the current of the system, maximizing the production of gas with respect to the power input, and giving higher process efficiency.

The application of pulsed power technology for hydrogen generation has been reported in the literature, including some improvements in the energy efficiency of the electrolysis process. In an investigation based on an inductive pulse generator circuit to supply the power to the electrolysis cell [21], it was shown that it is possible to increase the source voltage without reducing its efficiency, or resistive losses, which directly increases the energy density of the device. In another work on pulsed electrolysis [22], conclusions were drawn about the influence of the alkalinity of the electrolyte. With a supply voltage of 1 V it was possible to obtain pulses of hundreds of volts between the electrodes, and the peak value of this pulse increases as the conductivity of the electrolyte decreases. With a lower resistance, the power is reflected in a higher current instead of a high voltage. In addition, it was shown that for pulses with a duration of less than 1 μs, the cell behaves like a high capacitance capacitor, where the charging and discharging behavior turns out to be dependent on the conductivity of water.

### 2.3. High Voltage Electrolysis

The application of a high voltage to the electrolysis cell is an area that has not been frequently studied, although there are some authors who have worked on this topic in recent decades [9,10,23,24,25,26].

In one of these studies a continuous voltage of 200 V DC was applied to an electrolyzer. With this voltage, there was an increase in temperature that made the liquid boil in the vicinity of the reaction, together with an increase in the production of hydrogen and oxygen. When the excitation of the charges reached sufficient energy to jump to the gaseous medium formed, a plasma discharge occurred; because of this, the current decreased drastically without increasing the production of gas. The temperature of the electrolyzer stabilized at around 90 °C, entering the denomination of cold plasma. The formation of plasma brings with it the generation of high temperatures at the site of the reaction, in this way, efficiency characteristics of high-temperature steam electrolysis can be obtained [14], which exceeds the efficiency of low-temperature alkaline electrolysis.

This situation occurs at voltage levels that can vary between 200 V and 800 V [9,27], depending on the concentration of additive in the electrolyte and the separation between electrodes. Plasma electrolysis has a great advantage over low-voltage electrolysis. When a stable plasma is formed in the reaction, drastic decreases in the current circulating through the cell can be observed, without decreasing the flow of gas produced. At high voltages, gaseous media behave as good electrical conductors, so electrons circulate through the gas present between the electrodes, preventing circulation through the electrolyte that offers greater resistance. This situation shows that the production of hydrogen can have characteristics that do not respond to the amount of charge applied to the electrolyte as proposed, new phenomena arise related to the decomposition of water by the force of the electric field that requires attention. In a study on pulsed electrolysis [19], it was found to be possible to use voltage pulses at a sufficiently high frequency to produce plasma in the electrolyte. With this technology, high energy densities can be achieved for short periods of time allowing for the production of generators with a higher power or reduced dimensions.

These technologies open the doors to the investigation of a new electrolyzer system. The system is simple to implement since it does not require complex materials of high thermal resistance or materials that are difficult to acquire. The system can also be made at a low cost due to its small size and high efficiency. There is the possibility of increasing the power and frequency beyond the levels studied previously, where frequencies around 200 kHz [28] and voltages of 140 V [21] have been reached, seeking to reach the point of making stable plasma formation viable. Achieving this goal can make it possible to implement hydrogen-generating devices for domestic purposes, such as in energy storage and transportation.

## 3. Hydrogen Generator

### 3.1. Circuit

**Figure 2:**
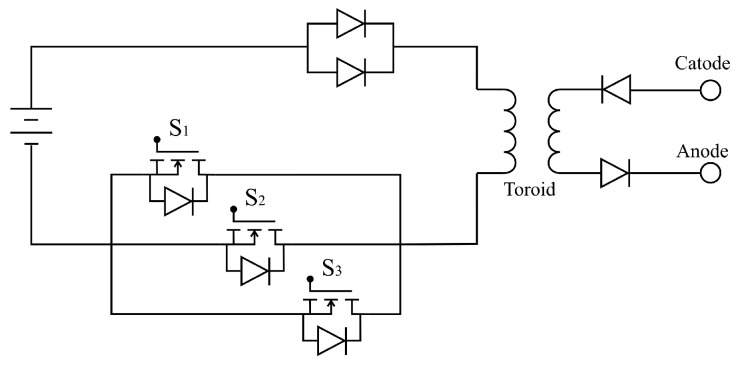
Schematic circuit of the inductive pulse generator.

The inductive pulse circuit shown in Figure 2, based on previous works [21], has a coil that is magnetized when the three switches close allowing current to circulate in the primary circuit. At the moment when the switches change state, the circuit opens allowing the abrupt discharge of the inductor.

The only path available for the energy stored in the magnetic field with this topology is in the secondary circuit circulating through the electrolysis cell. Three MOSFETs were used in parallel to reduce the impact of the current causing heat in the semiconductors. In addition, the equivalent resistance of the circuit is decreased because in conduction mode each switch has a certain internal resistance. The current of an inductor is given by:

(2) $$i_{L}\left(t\right)=i\left(t_{0}\right)+\frac{1}{L}\int_{-\infty }^{t_{0}}V\left(t\right)\;dt.\;$$

From this equation it can be seen that the current increases in the form of a ramp over time, inversely proportional to the magnitude of the inductance and the magnetic field formed is proportional to the current that circulates in the coil. To work with high frequencies, a relatively low impedance is needed, so the current can reach a significant value in a short time, allowing a complete discharge of the power stored in each cycle.

The parallel diodes located at the positive terminal of the power supply fulfil the function of protecting both the source and the MOSFETs from the peak of inductive voltage produced in the inductor at the time of opening the circuit. The voltage is presented at both ends of the toroidal transformer, but the current only has output through the secondary circuit, so it is necessary to prevent the semiconductors from seeing a pulse that exceeds their breakdown voltages.

**Figure 3:**
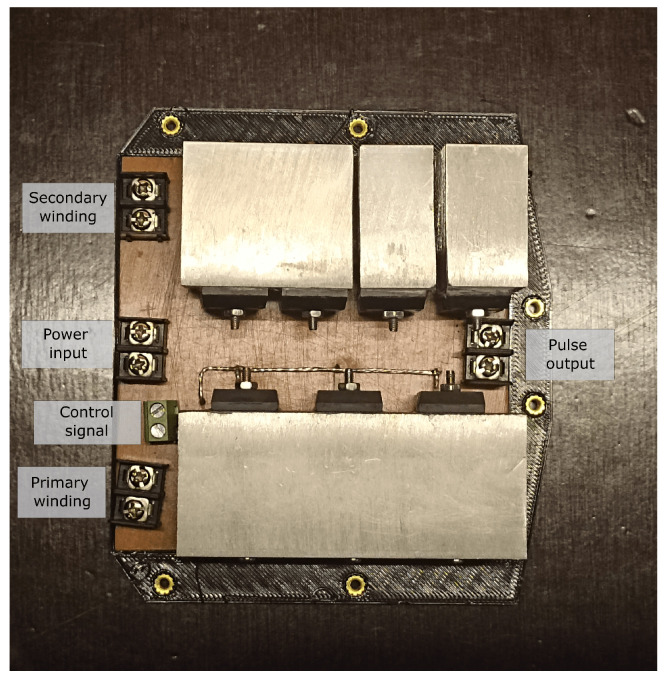
Assembled pulse generator circuit.

The function of the diodes in the secondary circuit is to prevent the circulation of reverse currents, allowing for the passage of voltage pulses with a defined polarity. The construction of the circuit was carried out on a copper plate where only the solid-state semiconductors were located (Figure 3). The coils, signal generator and actuator driver were implemented separately to obtain a modular design.

It is possible to achieve a pulse-generating device that delivers a stable and set voltage, in addition to offering greater control over the waveform. This can be achieved with a capacitive discharge. This type of circuit implies a greater degree of complexity in the manufacture and control, specifically in relation to the implementation of a high-voltage capacitor capable of storing the voltage necessary for the formation of plasma, another way to achieve this is stacking a series of capacitors to reach the necessary voltages. The idea is to control the voltage of the stored energy using a boost converter and then discharge the capacitors abruptly using MOSFETs as switches. This is left for future research due to the limited resources and time of the present study, for the purpose of studying the possibility of plasma formation and the molecular dynamics of the decomposition on water under high, inductive voltages pulses offers suitable parameter to start an investigation.

### 3.2. Plasmolysis Cell

**Figure 4:**
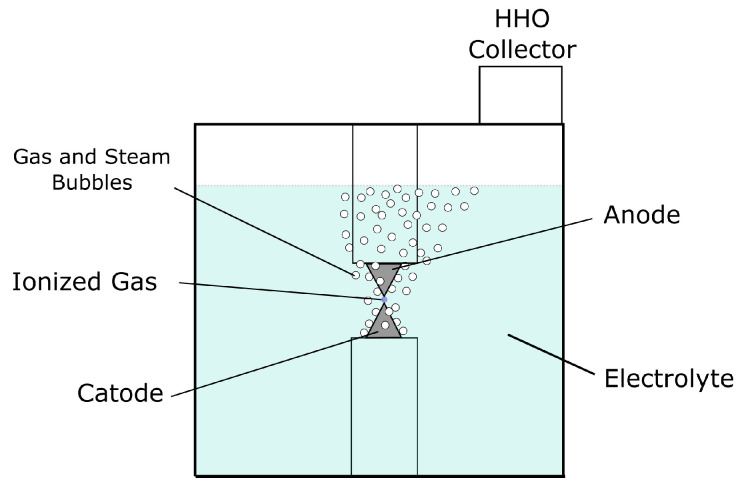
Pulsed plasmolysis cell diagram (front view).

**Figure 5:**
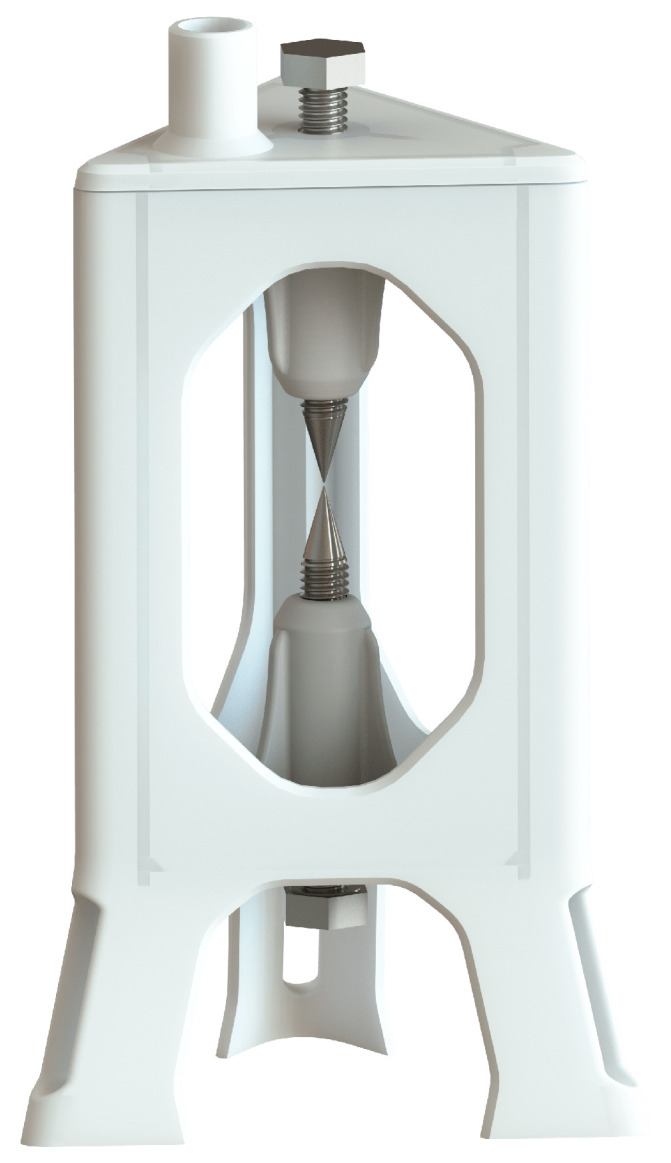
Pulsed plasmolysis cell render.

A pulsed plasma electrolysis cell has the same components as a conventional electrolysis cell, the main differences being the arrangement and size of the electrodes (Figure 4 and Figure 5). In this case, the electrodes have a needle-tip shape, which limits the surface area to a minimum, reducing the electrical capacitance to work with high frequencies, and also concentrating the power of the electric field at a specific point. Plasma formation requires high energy density, and the geometry of the electrodes influences the shape of the electric field that occurs between two conductors of opposite polarity.

With an electric field concentrated at a small point, it is expected to locate a large production of gas, which would be the medium for plasma formation. The frame of the cell built for this study is made with a UV resin used in 3D printing with LCD technology. This resin is able to withstand temperatures around 200 °C. There are three windows to observe the reaction inside the cell, constructed of glass with a thickness of 4 mm fixed to the base with structural silicone. The electrodes correspond to stainless steel bolts fixed by an internal thread printed as part of the frame of the cell, the end is sanded in the form of a needle tip. To adjust the separation between electrodes, the bolts can be turned to the appropriate position.

## 4. Experimentation Method

### 4.1. Circuit

The response of the circuit used in this study was analyzed with a Keysight DSOX1102G oscilloscope. In addition, this instrument allows the control of a signal generator for the actuation of the gate driver.

**Figure 6:**
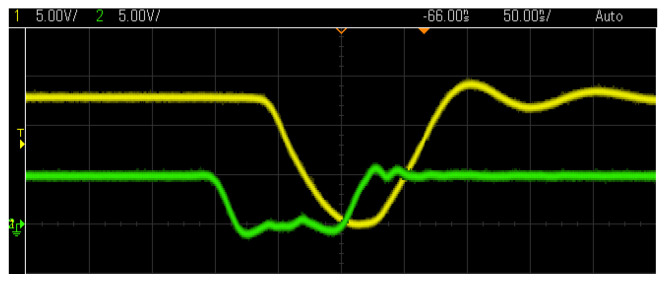
Estimated response of a 100 ns pulse.

Figure 6 shows the control signal next to the driver’s response for a 100 ns pulse. The yellow curve represents the output of the gate driver, while the green line represents the actuation of the control signal for a pulse of 100 ns. Both signals have different voltage levels, depending on the device under control. For the gate driver, a 5 V signal is used. The signal coming from this has the purpose of switching the state of the MOSFET which has a voltage of around 12 V in its high state. A delay of approximately 20 ns can be seen after receiving the state change command. The driver switching times are about 50 ns each (fall-time and rise-time). For a pulse of 100 ns, the gate driver delivers a signal of approximately 30 ns in the low state, making the pulses shorter than demanded.

**Figure 7:**
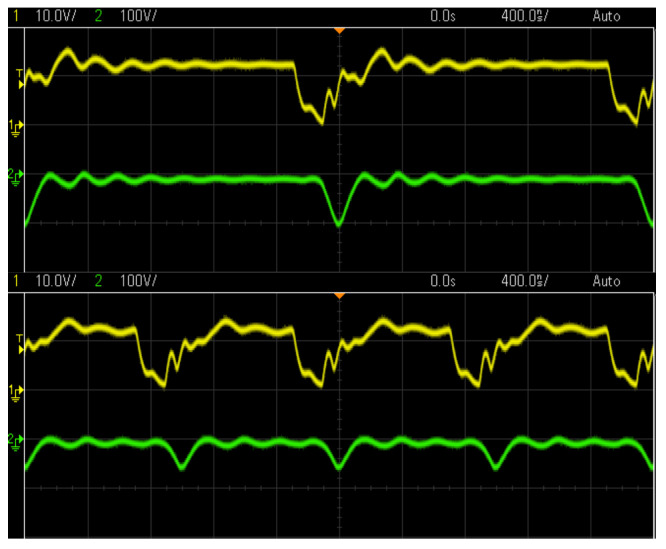
Voltage pulses obtained with voltage of 4 V, a separation of 1 mm between electrodes, frequency of 500 kHz (upper) and 1 MHz (lower) in drinking water. The yellow line represents the action of the driver and the green line the voltage in the cell.

The behavior of the circuit is mainly defined by the nature of the inductor and the frequency of the pulses. With a low repetition frequency, and a pulse duration of around 200 nanoseconds, the inductor has a longer charging time per cycle and more energy is accumulated compared to the same situation with a higher frequency. Figure 7 shows this situation: a comparison of the response of the cell against two different frequencies is shown, the pulse duration configured in the driver is exactly the same for both, and in the lower graph the frequency is set to twice the upper graph. When the inductor has exactly half the time to gather energy, it results in half the voltage in the pulse.

The oscillation in the voltage of the cell after the application of the pulse is related to the response of an RLC circuit, the secondary winding of the transformer forms a circuit of this type due to the capacitive and resistive characteristics of the cell. When working with frequencies above 1 MHz, the MOSFETs generate significant amounts of heat and the resolution of the wave decreases. The square wave utilized for control starts to look similar to a sine wave, which fails to commute the semiconductor correctly. To prevent damage to the device, these frequencies were not utilized.

### 4.2. Measurement System

**Figure 8:**
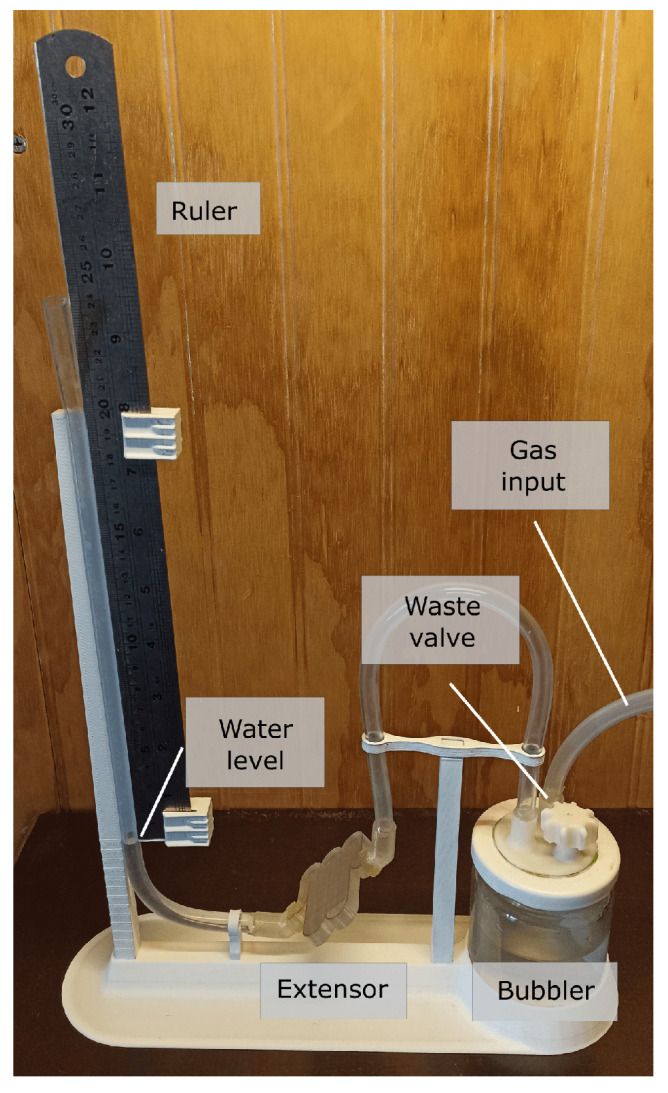
Assembled measurement system.

**Figure 9:**
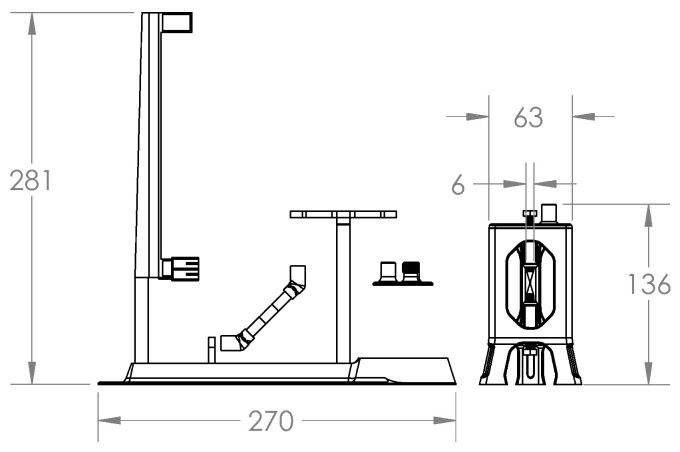
Measurement system and cell schematics.

The volume of gas used for the calculations is the totality of the gas produced in the decomposition reaction of the water molecule, two atoms of hydrogen and one of oxygen. This gas that contains both elements is called oxyhydrogen or HHO. The accumulated gas pressure will cause an increase in the height of the water column inside a transparent hose, this increase will be measured by a ruler located parallel to the hose containing the water, similar to a simple water level (Figure 8 and Figure 9). After taking the data, the pressure is released through a waste valve located in the bubbler lid. To obtain a characterization of this cell, its response to a series of frequencies, voltages and separation between electrodes will be reviewed. The electrolyte with which the main characterization can be carried out with drinking water without additives.

The first series of tests performed on the electrolysis cell consists of varying the frequency of the voltage pulse. The voltage of the power source is kept constant, as well as the separation between electrodes. The production was calculated with the power delivered by the power source. The efficiency of the process is evaluated without analyzing the efficiency of the circuit itself.

### 4.3. Electrolyte

To enable plasma formation in the plasmolysis cell using the inductive pulse circuit, it is necessary to minimize the electrolyte conductivity to achieve high-voltage pulses. The electrolyte with the lowest conductivity is distilled water, which, unfortunately, results in low efficiency in water decomposition.

Once the system is characterized using tap water, distilled water is used for the obtained high-voltage pulses. The measurements of efficiency made on this work can not be compared in the current form because it would be far less efficient than industry standards. We developed our prototype based only on the available components in our laboratory. These measurements are only relevant in the context of this study, relating the efficiency of pulsed electrolysis in equal conditions for the parameters presented.

### 4.4. Results and Discussion

**Figure 10:**
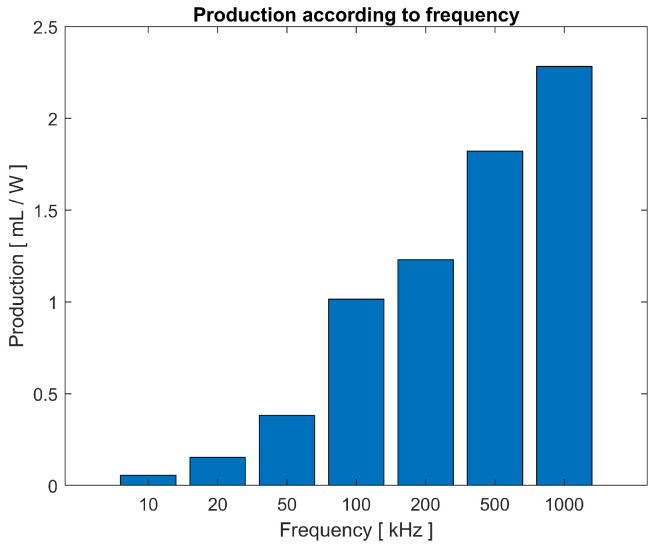
Gas production according to applied pulse frequency, with 4 V at the source and 0.1 mm separation between electrodes.

In the bar graph shown in Figure 10, the production of HHO in milliliters per watt of power in relation to the pulse rate are shown. A clear trend of increase in the efficiency of the process can be seen as the frequency is increased. This information allows us to optimise the operating frequencies.

**Figure 11:**
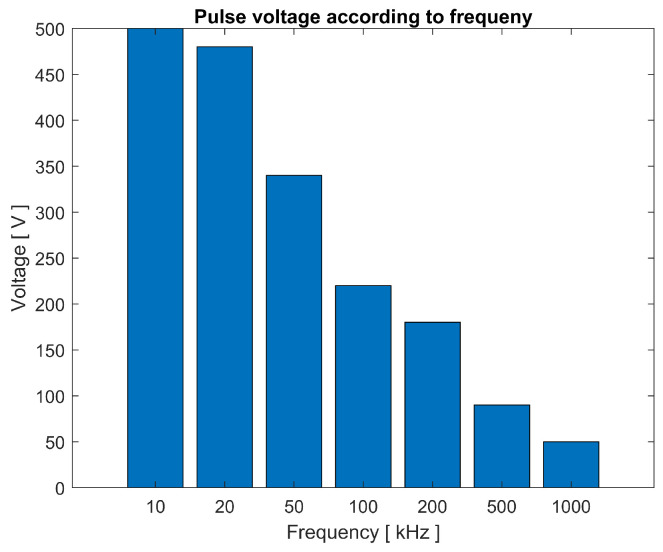
Pulse voltage obtained according to application frequency, with 4 V at the source and separation of 0.1 mm between electrodes.

By observing the pulse voltage data, obtained with the previous experiment, maintaining the separation between electrodes of 0.1 mm and a voltage of 4 volts at the source, a variation in the maximum voltage of the pulse that responds inversely proportional to the increase in frequency can be seen in Figure 11.

The behavior of the circuit is mainly defined by the nature of the inductor, and the experiment varies the frequency of the pulses. However, the duration of these is maintained. When a low frequency in the repetition of pulses with a duration of around 200 nanoseconds is observed, the inductor has a longer charging time per cycle and more stored energy compared to the same situation with a higher frequency. When the inductor has exactly half the time to gather energy it is obtained as expected, where half of the voltage is in the pulse; see Figure 7.

**Figure 12:**
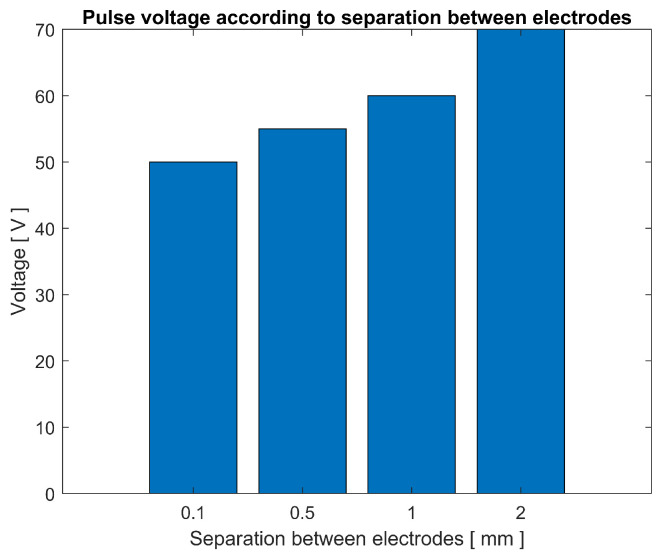
Pulse voltage obtained according to separation between electrodes, with 4 V at the source and pulse frequency of 1 MHz.

Figure 12 shows a trend of voltage increase proportional to the increase in distance between electrodes. It can be seen that the pulse voltage depends partially on the resistance of the circuit. When the energy accumulated in the inductor in the form of a magnetic field is abruptly discharged, and the circuit has low resistance, the power can flow with a high current, the voltage varies its intensity according to the current to maintain the power ratio.

If the distance that the current must travel inside the electrolyte is increased, the resistance of the circuit is directly increased, resulting in a higher voltage, unlike when the electrodes are closer to each other or in a lower resistance configuration. After reviewing the influence of frequency and resistance in the process, the possible effects of capacitance are introduced, which in an environment dominated by frequencies tends to produce resonance phenomena [20].

The electrolysis cell has a composition that behaves similarly to a capacitor, and there are two electrodes separated by an electrolyte. The capacitance in these components increases as the surface area of the electrodes grows and the separation between them decreases. In this case, the electrodes were optimized to concentrate the electric field with a needle-tip shape, leaving only one variable to control the capacitance of the cell, the separation between electrodes. Following this analogy, if the electrodes move away, the electric capacitance decreases, changing the resonant frequency of the circuit. A capacitor with a lower capacitance has a shorter charge and discharge time, therefore it is able to work at higher frequencies without maintaining a constant voltage.

**Figure 13:**
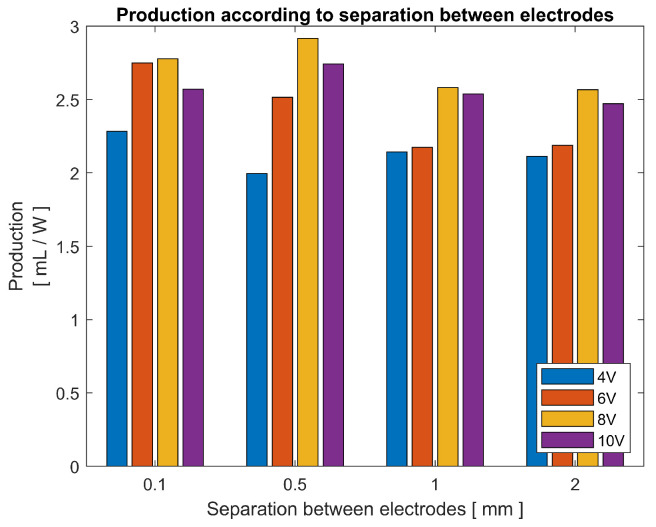
Gas production according to separation between electrodes, with a variation of 4 to 10 V in the power source and pulse frequency of 1 MHz.

Figure 13 shows the production in relation to the separation between electrodes along with a variation in the voltages of the power supply in tap water. The graph starts with a minimum of 4 V, when using lower voltages, the production is too low to obtain comparable results. For 4 V and 6 V voltages, the highest output is found when the electrodes are very close to each other, as they move away production decreases.

It is different in the case of higher voltages, for 8 V and 10 V the production peak is at 0.5 mm, a slightly bigger separation. The fundamental difference between the pulsed electrolysis process with conventional electrolysis is that by applying extremely fast pulses, the current is able to enter the electrolyte before the breakdown of the water molecule occurs, and the decomposition continues until after the pulse is finished. However, it is thought that depending on the electrolyte, there is a limit to how much energy can be entered and chemically accumulated in the liquid.

A distinction must be made between two capacitive behaviors present in the electrolysis cell. One is the ability to store or accumulate chemical energy in the electrolyte; after the pulse, this energy is used to break down the molecules of H_2_O, this will be referred to as chemical capacity. The other capacitive behavior is the physical property that two conductors have to condense electrons on the surface under a potential difference, increased by the electrodes positioned very close to each other and is diminished when using needle-tipped electrodes, this principle is the cause of the double layer effect, referred to as electric capacitance.

Applying the theory of the capacitor to the electrolysis cell involved in each voltage pulse, a certain amount of current is injected into the cell; this current is greater when the voltage is greater. Taking into account that the gas or decomposition of H_2_O is produced in the vicinity of the electrodes, a saturation of the medium in which the current is applied, in this case, water, can be proposed. In this way, by increasing the separation of the electrodes, the area available to collect charge is expanded, or it could be related to a volumetrically increased chemical capacity. Separating the electrodes brings with it other effects, in addition to increasing the chemical capacity, the electrical capacitance is reduced and also increases the resistance of the circuit, which causes the pulse voltage to increase also for this circuit. The distance between electrodes must be carefully studied for the frequency and voltage to be applied in the electrolysis cell.

During tests with rainwater as an electrolyte, after the first stage of the experiment on drinking water, high levels of voltage in the pulses were presented; a couple of hundred volts due to its low conductivity. Rainwater is similar in purity to distilled water, although it may contain minerals or particles corresponding to environmental conditions. It has a lower ionic concentration that reduces its chemical capacity compared to drinking water. Around a frequency of 500 kHz, a voltage of 10 V in the power supply and with a separation of 0.1 mm between electrodes, pulses of 430 V were reached, which caused the formation of electrical sparks in the vicinity of the electrodes.

This phenomenon was not present constantly, it was exhibited intermittently as small bubbles were formed that were adhered between the tips of the electrodes, a location conducive to the ionization of the gaseous medium, by further decreasing the distance between them, it was possible to evidence the formation of plasma.

**Figure 14:**
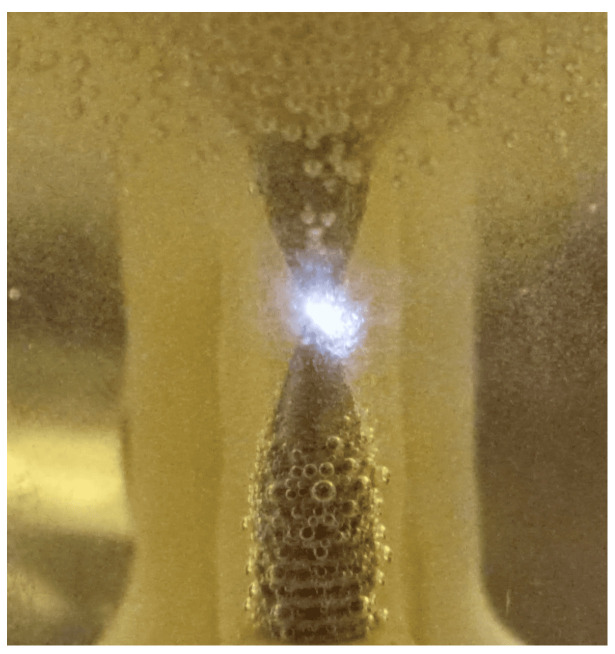
Plasma formation inside the electrolysis cell.

At the time of plasma formation (Figure 14), it can be noticed that gas bubbles begin to be produced in greater proportion directly from the ionized gas zone. Unfortunately, it was not possible to stabilize the phenomenon, the electrodes had to approach a distance of less than 0.1 mm to stabilize it for a few seconds, after a while it became intermittent, this behaviour can be explained for the disintegration of the electrodes under high energy densities [23].

Due to this, it was not possible to carry out the corresponding gas production efficiency tests under plasmolysis. Despite the drawback, this experiment allowed information on the needs that must be covered for the formation of stable plasma to be obtained. The pulses must be above a voltage of approximately 500 volts for this distance (less than 0.1 mm), in this way the electric arc may be viable at a greater distance between electrodes, the voltage must increase proportionally to the distance.

For the formation of a plasma in any electrolyte to be possible, it is necessary that the pulse-generating circuit is capable of producing these with a voltage independent of the resistance of the liquid medium. The latter can be achieved by performing abrupt and intermittent discharges from a high-voltage stabilized direct current source, possibly a high-voltage capacitor. Current technology allows for the implementation of solid-state voltage boosters that could be suitable for this application.

There are other alternatives for improving efficiency in this field, such as the implementation of more complex topologies to form the voltage pulses, such as inductive adders or the use of a Pulse Forming Lines (PFL) [29,30]. These methods can significantly enhance the pulse parameters.

## 5. Conclusions

Pulsed power electrolysis offers the ability to improve the efficiency and production of the process. With these advantages, it is possible to increase the power density of electrolysis devices allowing for the development of portable applications.

The efficiency of pulsed power electrolysis depends on a marked separation between voltage pulses. If a constant or quasi-constant voltage is maintained within the cell, without the potential being neutralized between pulses, the process takes on the characteristics of conventional electrolysis causing the formation of the double layer.

The formation of plasmolysis was achieved at a frequency of 500 kHz and a pulse voltage of 500 volts in distilled water. To work with more efficient electrolytes, the application of pulses from a stable high-voltage source capable of delivering a certain voltage independent of the purity of the electrolyte is proposed.

## Data Availability

Not applicable.
